# Adding volition to word processing: Expected utility norms for 80,000 English words and multiword expressions

**DOI:** 10.3758/s13428-026-03051-8

**Published:** 2026-05-27

**Authors:** Andrew Wang, Marc Brysbaert, Fritz Günther

**Affiliations:** 1 Melbourne School of Psychological Sciences, University of Melbourne, Melbourne, 3010 Australia; 2 Department of Experimental Psychology, Ghent University, Ghent, Belgium; 3 Department of Psychology, Humboldt-Universität Zu Berlin, Berlin, Germany

**Keywords:** Expected utility, Word recognition, Multiword expressions, Lexical decision

## Abstract

This study examined the concept of word usefulness by analyzing expected utility ratings for over 80,000 English words and multiword expressions. Participants used best–worst ratings to indicate how useful it is to know each word/expression. Our findings show a high level of agreement regarding the usefulness of words and expressions. Stimuli were rated as more useful if they were more frequent, widely known, learned early in life, and central to the semantic network. Concreteness had a substantial negative correlation, indicating that abstract words in general received higher utility scores than concrete words. Positive stimuli received slightly lower utility scores than negative stimuli. Expected utility was a good predictor of which words are known to speakers of English as a first and second language, but did not contribute to predicting response times to known words. These findings suggest that expected utility is a variable affecting which words are likely to be learned, but does not affect word processing times (much). The expected utility scores are freely available for research and education.

## Introduction

Some words are less likely to be known than others (Brysbaert et al., 2016b, 2019b). These tend to be words that rarely occur in a language (Brysbaert et al., 2016a, 2018), long words (Balota et al., 2007; Brysbaert & Drieghe, 2003), words with unusual spellings (Yarkoni et al., 2008), and words typically acquired later in life (Juhasz, 2005). Those words that are known to few people also take longer to recognize (Brysbaert et al., 2016b, 2019b).

Most differences in word processing effort can be interpreted in terms of cognitive processes involved in learning and memory retrieval. The frequency effect, for example, can be seen as the equivalent of the ubiquitous practice effect in learning, which increases the association strength between memory representations (Monaghan & Ellis, 2010) or increases the number of memory instances (Logan, 1988). The age-of-acquisition (AoA) effect can be understood on the basis of neural networks, where it is observed that items learned first are able to change the network to a greater extent than items learned later, when the knowledge already stored in the network must be preserved (Ellis & Ralph, 2000). Less efficient processing of words with unusual spellings can be understood within networks as a result of interactions between form recognition and meaning activation (Grainger & Jacobs, 1996). The word length effect can be related to the amount of information present in the stimulus (Lewis & Frank, 2016; Piantadosi et al., 2011).

A characteristic of all existing interpretations is that they assume the human brain to be the equivalent of a twentieth-century computer, a machine differing only in memory capacity and processing speed, unaffected by the utility of the information received. As such, the existing interpretations are a nice illustration of the impact the computer metaphor has had on cognitive psychology (Cisek, 1999).

A basic prediction of the twentieth-century computer metaphor is that word processing should be the same for all stimuli. Therefore, the word frequency effect should be the same for all words or can be explained by interactions with other word-specific features, such as an interaction between word frequency and word length. Research on word knowledge does not agree with this basic prediction, however. Words like “airbag,” “unicorn,” and “evaporate” are recognized as fast and accurately as high-frequency words according to the English Crowdsourcing Study (Mandera et al., 2020), despite the fact that their frequency of occurrence in the English language is low. Similarly, some of the best-known words by second language (L2) speakers of English are low-frequency words such as “midnight,” “popularity,” and “sexy” (Brysbaert et al., 2021).

One factor that could be involved in words that are easier to process than expected is that the frequency of some words in everyday life has been underestimated, because the language corpus on which word frequency estimates are based does not include all language registers. Although this factor is likely involved, it seems unlikely to explain everything, given the diversity of words that are recognized better than predicted on the basis of frequency. A second factor could be that the word frequency effect is not the result of a practice effect, but due to a memory network, which promotes access to words used in multiple different contexts over words encountered in a small number of contexts (Chang et al., 2023; Johns, 2024). According to this interpretation, words such as “airbag,” “unicorn,” “evaporate,” “midnight,” “popularity,” and “sexy” are well known because they are useful in more contexts than words of the same length and frequency that are more difficult to process, such as “piñata,” “putrid,” “reminisce,” “incident,” “redemption,” and “firm.”

A third explanation could be that not all words are learned and retained in memory equally well. People learning a second language are familiar with this experience. Vocabulary acquisition is one of the main obstacles in acquiring a new language. Words memorized for an exam are often forgotten a few weeks later. At the same time, other words seem to stick, even though the exposure to them was in no way higher than that to other words, rather the opposite. Some words seem to be acquired after a single exposure (Rice et al., 1990); other words never seem to be mastered despite being encountered in the language to the same extent, such as “furfural,” “calomel,” “brogan,” or “cully.” Why would this be?

A basic concept in economics is "subjective expected utility" (Fishburn, 1981), the expected attractiveness of pursuing a particular option. Could it be that some words are more likely to be learned and retained because they are perceived as more useful than others?^1^ There is some evidence pointing to this possibility. One finding is that people are more likely to remember information if shortly before they failed to answer a question related to that information (Brod, 2021; Kornell, 2014; Pan & Carpenter, 2023). Several variables have been proposed to account for this learning enhancement, including increased motivation, interest, attention, surprise, and curiosity. This is how Brod (2021, p. 1843) speculated about how surprise can improve information processing: “Surprise as measured by pupil dilations is closely coupled with the release of norepinephrine in the brainstem’s locus coeruleus … Release of norepinephrine by the locus coeruleus influences arousal levels and has been shown to prioritize processing of goal-relevant information and to promote memory formation in the hippocampus.”

Other evidence that expected utility may influence word processing comes from L2 learning. He and Godfroid (2019) collected measures of word frequency, word difficulty, and word usefulness for 191 English academic words and multiword expressions. Word difficulty was rated by English L2 teachers who were asked to indicate how advanced the vocabulary was (1 = basic/easy, 7 = advanced/difficult). Word usefulness was estimated by asking the same teachers how worthwhile knowing the words would be to students preparing for college in North America (1 = not worthwhile, 7 = indispensable). Brysbaert et al. (2021) correlated the measures with word knowledge in speakers of English as L2. The correlation was highest for word difficulty (*r* =.60), followed by word frequency (*r* =  −.52). However, word usefulness also correlated with word knowledge (*r* =  −.47), and multiple regression indicated that all three variables contributed significantly to the prediction of L2 word knowledge (*R*^2^ =.46).

The concept of expected utility also closely relates to the notion of core vocabulary, the set of words which are the most important, central, or fundamental in a language (Bell, 2012; Carter, 1998; Hulstijn, 2024; Lee, 2001; Stubbs, 1986; Wang et al., 2025, 2026). There are many possible ways to define core vocabulary, including the semantic primitives which are sufficient for defining all other word meanings (Wierzbicka, 1996), those words which all native speakers know, definitions using structural-semantic criteria (Carter, 1998; Lee, 2001), items which are resistant to borrowing from a diachronic perspective (Zenner et al., 2014), and words which follow consistent patterns of use cross-linguistically (Calude & Pagel, 2011, 2014), among others. Recent work has begun to investigate core words from a psycholinguistic perspective (Wang et al., 2025, 2026), linking the concept of how useful or important words are back to how people represent, process, and use language as a starting point. In this view, the core words in language can be captured by lexical factors such as frequency, AoA, semantic centrality, use across different contexts, and perceived utility.

To investigate whether expected utility affects language processing on top of established variables, we need information about the variable. In addition, it would be good to have expected utility norms for multiword expressions as well as words. It has become clear that norming has been centered too much on individual words, whereas a large part of language consists of multiword expressions. These include compound nouns, particle verbs, fixed expressions, and idioms. Muraki et al. (2023) compiled a list of 62,889 English multiword expressions, which is larger than the list of 61,853 English words used in the largest English vocabulary study so far (Brysbaert et al., 2016a).

Muraki et al. (2023) collected concreteness ratings for the multiword expressions and Mohammad (2026) collected anxiety associations for 20,000 of them. However, these are the only human-provided data currently available. Other information comes from artificial intelligence (AI)-generated estimates. Brysbaert et al. (2025) obtained estimates of familiarity, and Martínez et al. (2025) provided estimates of concreteness, valence, and arousal. It is essential to have more human-provided information about expressions in addition to individual words.

## Method

### Materials

The selection of materials began with the lists of words and multiword expressions used by Brysbaert et al. (2016a) and Muraki et al. (2023). To these, we added more than 13,000 new (hyphenated) words and expressions collected since the publication of the lists. Because expected utility scores are only interesting for stimuli that people are familiar with, we removed all words/expressions with a large language model (LLM)-generated familiarity score of less than 3.5 on a scale of 1 (completely unfamiliar) to 7 (completely familiar), since those words are unlikely to be known by 90% of people. These familiarity scores were generated by querying LLMs (specifically, GPT-4) with familiarity rating instructions for an extensive range of words and expressions. Research by Brysbaert et al. (2025) showed that LLMs can provide useful familiarity estimates on the basis of their training data. These familiarity estimates are highly correlated with existing human familiarity ratings such as the Glasgow norms (Scott et al., 2019) at *r* =.76, similar to correlations observed between different sets of human familiarity ratings themselves. Although the procedure of excluding words/expressions with familiarity estimates below 3.5 carries the risk of excluding some interesting stimuli, it saved us from collecting data for 50,000 uninteresting stimuli, as word familiarity is the best variable for determining which stimuli are known to people (Sendín et al., 2025). Too many unfamiliar words/expressions can also be confusing for participants, as they may wonder how to assess the usefulness of stimuli with which they are unfamiliar. The pruning resulted in a total of 82,880 entries, making for the largest list of stimuli presented to human raters so far. Of these, 34,893 were words and 47,987 were multiword expressions.

### Participants

Participants were recruited through Prolific (https://www.prolific.com/), a company that connects researchers with paid participants. A total of 6,186 participants were tested, of whom 5,526 provided usable data (see below for the exclusion criterion). The ages of the 5,526 selected participants ranged from 18 to 93 years (*M*_age_ = 44.9 years, *SD*_age_ = 13.6 years). Among participants, 2,985 self-identified as female, 2,480 as male, 38 as nonbinary, and 23 indicated the category “other.” The study took on average 12 min, for which participants received £2. Participants had to be over 18 years of age, reside in the USA or UK, and have an approval rating of 95–100% and at least 500 previous submissions. They had to be monolingual native English speakers and could only participate in the study once. In the final sample, 55.5% of participants were from the USA, and 44.5% were from the UK.

### Procedure

Testing was conducted online, with the use of jsPsych (de Leeuw et al., 2023). Participants were informed that they would see a variety of English words and expressions. They were asked to indicate how important/useful it was for someone to know each stimulus. After being informed about the purpose and the nature of the study, participants were asked to consent for their data to be analyzed anonymously and shared as part of a scientific publication.

Participants who gave informed consent were subsequently told that they would see 50 trials. On each trial, a sample of six words and multiword expressions were shown. Participants were asked to indicate which of the words/expression they found the most useful to know and which the least useful, following the best–worst procedure described by Hollis (2018, 2020). If a word/expression was unknown to the participant, they were asked to assign it to the least useful stimulus for them. The instructions provided to participants were as follows:

Some English words/expressions are more useful to know than others. How important/useful do you think it is for someone to know each word/expression? On each trial you will see a set of 6 words/expressions. Please select which word/expression you think is the most useful of the set and which you think is the least useful. The question is not how useful the thing described is, but how useful it is to know the word/expression. Sometimes the choices will be obvious. Sometimes the choices will be difficult. We rely on your judgment to help us decide which words/expressions are useful to know or learn.

For example, if you think "hammock" is the least useful word/expression to know among the set, select it as the least useful. If you think "give up" is the most useful word/expression to know among the options, select it as the most useful.

If there is a word in the list that you do not know, then this word has not been useful for you and you can select it as the least useful word.

Five of the 50 trials were catch trials that had clear best and worst answers, because one alternative was very frequent/prevalent and one alternative was unlikely to be known to the participants (all the worst catch trial items had prevalence values of less than 1% known). The catch trials were always the same five trials, one of which is shown in the following example:

- ask a question
- check mark
- unclouded
- multimedia system
- top edge
- yataghan

Of these stimuli, “ask a question” is arguably the most useful to know for participants and “yataghan” the least useful to know. The catch trials were pretested on an independent group of participants and checked after the first few hundred participants to make sure that the best and worst entries were selected with very high probability. Participants received a penalty of 3 if they chose the least important alternative as the most important and vice versa. If they chose one of the other four alternatives as the least or most important alternative, they received a penalty of 1. The penalties were added up over the five catch trials, and participants with a penalty score of more than 5 were excluded from the analysis on the basis of careless responding (or at least responding in a manner that was not in accordance with the instructions). The excluded participants were replaced with other participants. As indicated above, 10.7% of the participants had to be replaced.^2^ The catch trials not only served to identify careless participants but also made the task clearer to the participants, as 10% of the trials were easy trials.

Permutations were made from the complete list of 82,880 stimuli, using software provided by Hollis (2018). The words/expressions were arranged into sets of six, which formed the item sets to be presented. We avoided repeating any combination of two stimuli across item sets, so that each word/expression was compared to the maximum number of alternative word/expressions (see Hollis, 2018, Experiment 4). Each participant was presented with a unique list of items.

To present each individual item 18 times across all participants (identified as sufficient for the near-perfect recovery of ground-truth scales in a simulation study by Hollis, 2018), we created a total of $\frac{18*\mathrm{82,880}}{6} = \mathrm{248,640}$ unique item sets. These were divided into 5,526 unique lists of 45 item sets (equivalent to 45 × 6 = 270 words/expressions per list).^3^ In this design, each word/expression was directly compared to 90 different items across the entire item set. The permutations were made from the complete list of 82,880 stimuli in three batches, which were then further subdivided into six lists within each batch to be more easily distributed across participants. This allowed us to calculate utility scores after each batch of six observations per item. This made it possible to get an estimate of the reliability of utility scores as soon as we had two batches.

## Results

All data and analysis code are available at https://osf.io/4nwgx/, so that readers can check our analyses and, if necessary, improve them.

Participants’ best–worst choices per item (trial) generate the information that the “best” (= most useful) item is preferred over the other five items, and additionally that the four nonselected items are preferred over the “worst” (= least useful) item. On the basis of all the pairwise comparisons from all trials and item set arrangements, numerical scores of expected utility can be calculated (Hollis, 2018; Hollis & Westbury, 2018). While several parameter estimation algorithms are available, Hollis (2018) identified Value Learning as the best option, which we adopted here.

**Fig. 1:**
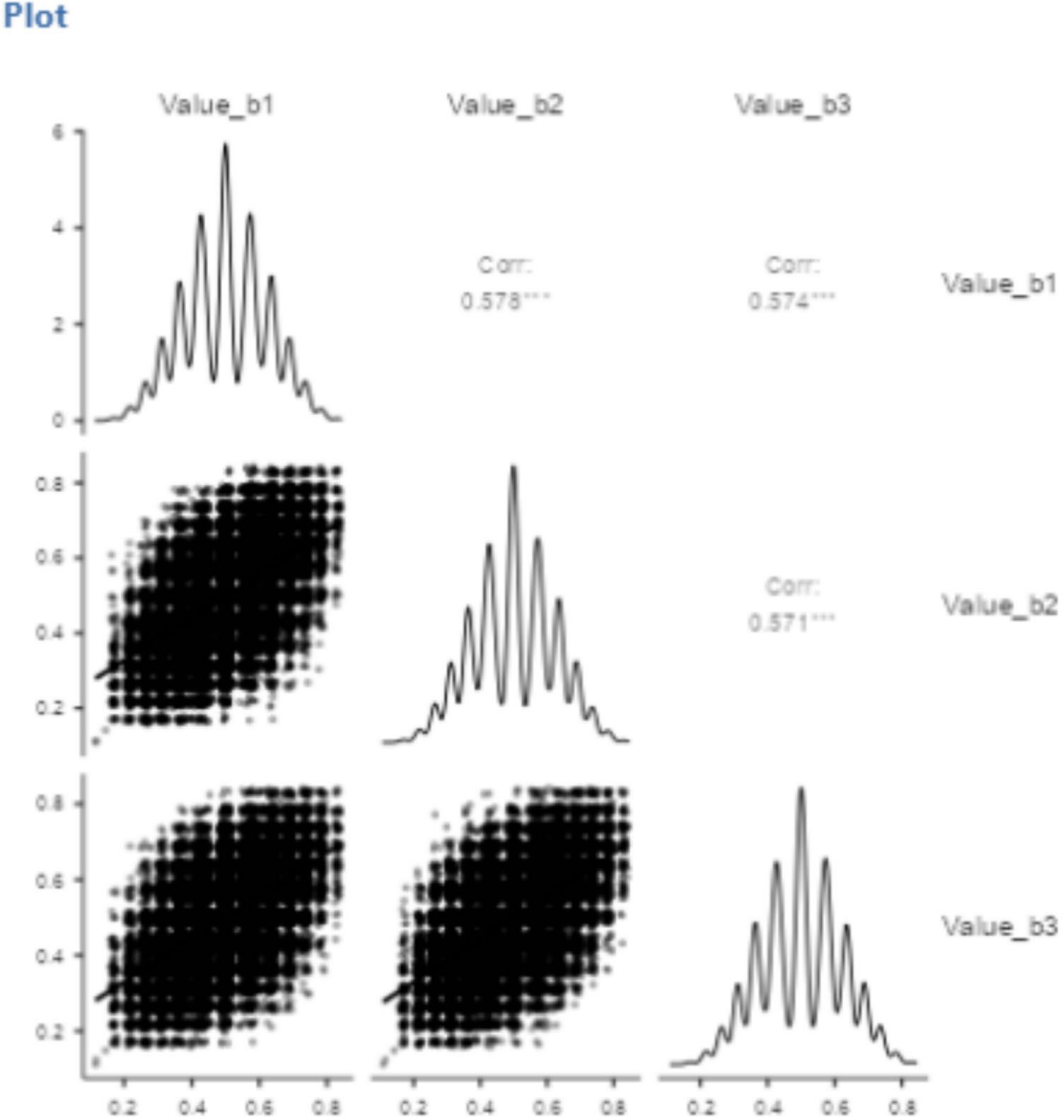
Distributions and correlations of the scores in the three batches of six evaluations per stimulus (figure created with ggcorrplot; Kassambara & Patil, 2023)

By presenting the items in three batches, we were able to calculate the utility scores after each batch. Figure 1 shows the distributions of utility scores in each batch and the correlations between the batches. Scores based on the Value Learning algorithm result in a near-Gaussian distribution of values between 0 and 1, with high values indicating words useful to know and low values words of less interest. Correlations between the batches were.57, resulting in a Cronbach’s alpha of.80 across the three batches. Figure 1 also shows that the granularity of scores based on six observations per stimulus is rather low. This is much less the case for scores based on the full 18 observations per stimulus (see Fig. 11), which is the score we used in all analyses below.

### Analysis of the word stimuli

To investigate the validity of the utility scores, we made a distinction between the words and the multiword expressions, because much more information is available for individual words than for expressions.

Table 1 shows the 20 words with the highest expected utility and the 20 words with the lowest expected utility. The three words with the lowest expected utility were those we used in the control items. This is partly because we used them to exclude participants who ranked them highly.

**Table 1:** Words with the highest and the lowest expected utility

Word	Utility	Word	Utility
happy	0.83	yataghan	0.11
important	0.81	witenagemot	0.12
hungry	0.81	stotinka	0.14
exactly	0.80	zeta	0.18
enjoying	0.80	mulatto	0.19
discussion	0.80	qubit	0.20
required	0.80	zinfandel	0.20
fine	0.80	matzah	0.20
beneficial	0.80	nom	0.20
immediately	0.80	oolong	0.20
alive	0.79	axon	0.20
information	0.79	lambda	0.20
grateful	0.79	operand	0.20
telephone	0.79	argon	0.20
explain	0.79	ester	0.21
life-threatening	0.79	sine	0.21
affordable	0.78	scalar	0.21
because	0.78	saran	0.21
sleep	0.78	syrah	0.21
disease	0.78	blitzkrieg	0.21

We compared the utility scores for words with the following variables that are known to influence word recognition and that are available for large numbers of words:

1. Word frequency: For this variable, we used the Multilex frequency norms (Brysbaert et al., 2025). These are mainly based on subtitles and are slightly better than SUBTLEX-US frequencies, because they are based on a considerably larger corpus. Frequencies are expressed in Zipf scores, which involves a logarithmic transformation (Van Heuven et al., 2014).
2. Word length, calculated as the number of letters in the word.
3. OLD20: The orthographic distance to the 20 closest words, as defined by Yarkoni et al. (2008), and taken from the SCOPE database (Gao et al., 2023).
4. Age of acquisition (AoA): Based on the Kuperman et al. (2012) norms.
5. Centrality in a semantic network (Assoc_Freq_Token123): Based on the log number of times the word is given as one of the first three associates across all cue words (De Deyne et al., 2019).
6. Word prevalence: The percentage of people who indicate they know the word in a yes/no decision task (Brysbaert et al., 2019b). Probit transformed.
7. Concreteness: Ratings from participants indicating how concrete the concept referred to by the word is, based on Brysbaert et al. (2014).
8. Dominant part of speech (dom_PoS), based on SUBTLEX-US (Brysbaert et al., 2012)
9. GPT estimates of familiarity: AI-generated word familiarity estimates from 1 to 7 (Brysbaert et al., 2025).
10. GPT estimates of valence and arousal: AI-generated estimates from 1 to 7, which according to Martínez et al. (2025) correlate most closely with the human ratings collected for smaller word samples.

**Fig. 2:**
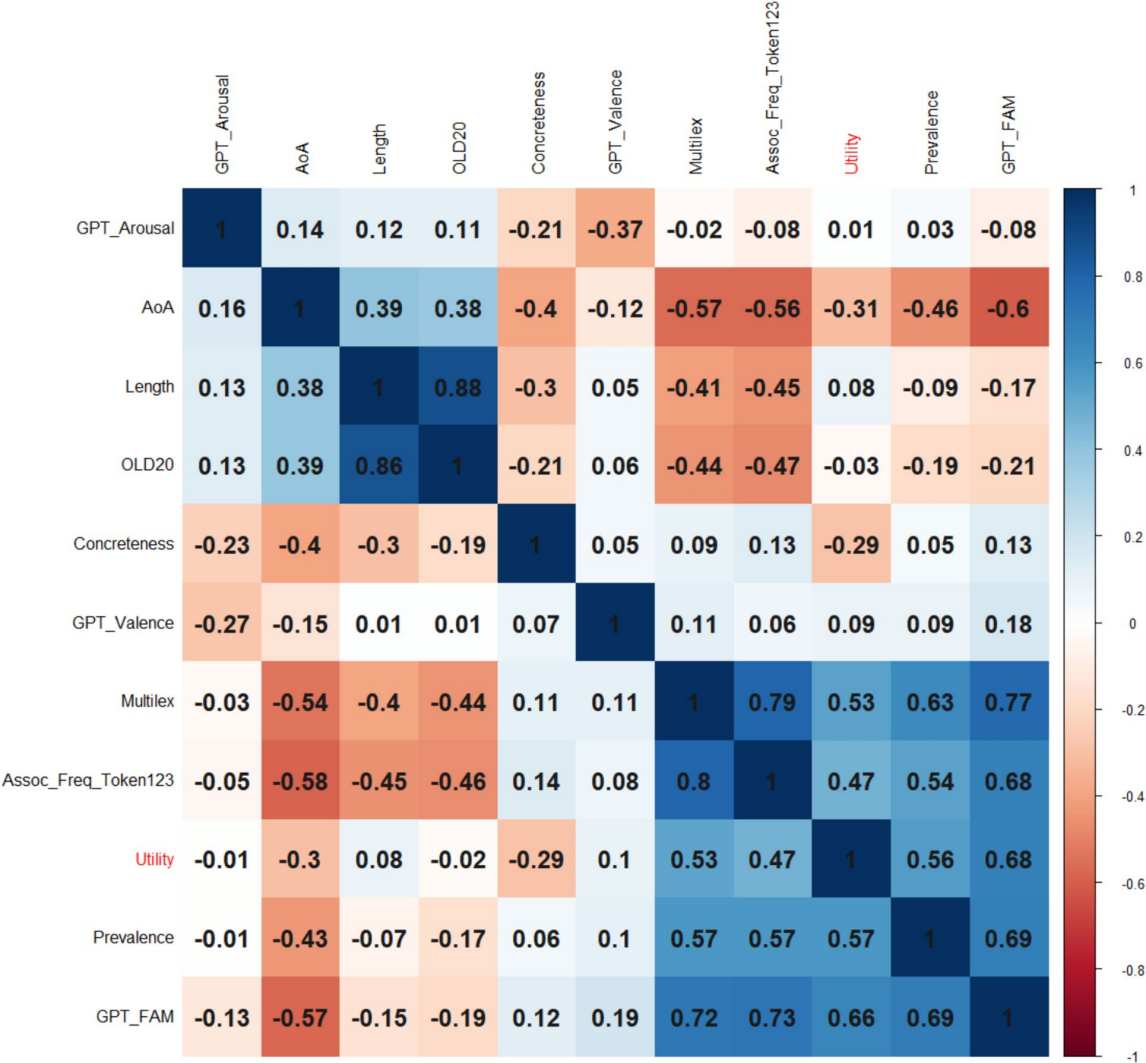
Correlations between the numeric variables, ordered according to a hierarchical cluster analysis (based on corrplot; Wei et al., 2017). Above the diagonal: Spearman correlations; below the diagonal: Pearson correlations

We had full data for 16,021 words, which was considered sufficiently representative not to delete a variable. In order to limit the analysis to content words, only words that had a dom_PoS of noun, verb, adjective, or adverb were retained (*N* = 15,704). Figure 2 shows the correlations between the variables (above the diagonal: Spearman correlations; below the diagonal: Pearson correlations). The order of the variables is determined by a hierarchical cluster analysis. Figure 2 shows that expected utility is part of a cluster with GPT familiarity, prevalence, centrality, and Multilex frequency.

**Fig. 3:**
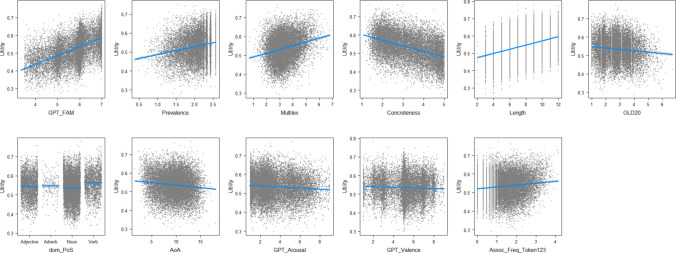
Predictors of expected utility (based on the visreg package in R; Breheny & Burchett, 2017)

To examine which variables influence utility, we ran a multiple regression analysis. This indicated that all variables were significant predictors (which is expected due to the large number of data points analyzed), together accounting for 63.1% of the variance. Figure 3 shows the effects of the variables. Expected utility increases as words become more familiar, more prevalent, more frequent, longer, and more semantically central. In contrast, expected utility decreases as words become more concrete, have higher orthographic distance to other words, are acquired at a later age, and are more positive and highly arousing. Expected utility is lowest for nouns, followed by adjectives, adverbs, and verbs.

To better gauge the relative importance of the variables, we ran a random forest analysis. This is a machine learning technique that combines the output of multiple decision trees to create a more accurate prediction. Each individual tree is built using a random subset of the data and a random subset of features, which helps to ensure that the trees are diverse and uncorrelated. We used the “H2O AutoML” R package (Lee & Gates, 2025) that automates the choices to be made, selects the most appropriate algorithm, and is optimized for the analysis of psychological data.

**Fig. 4:**
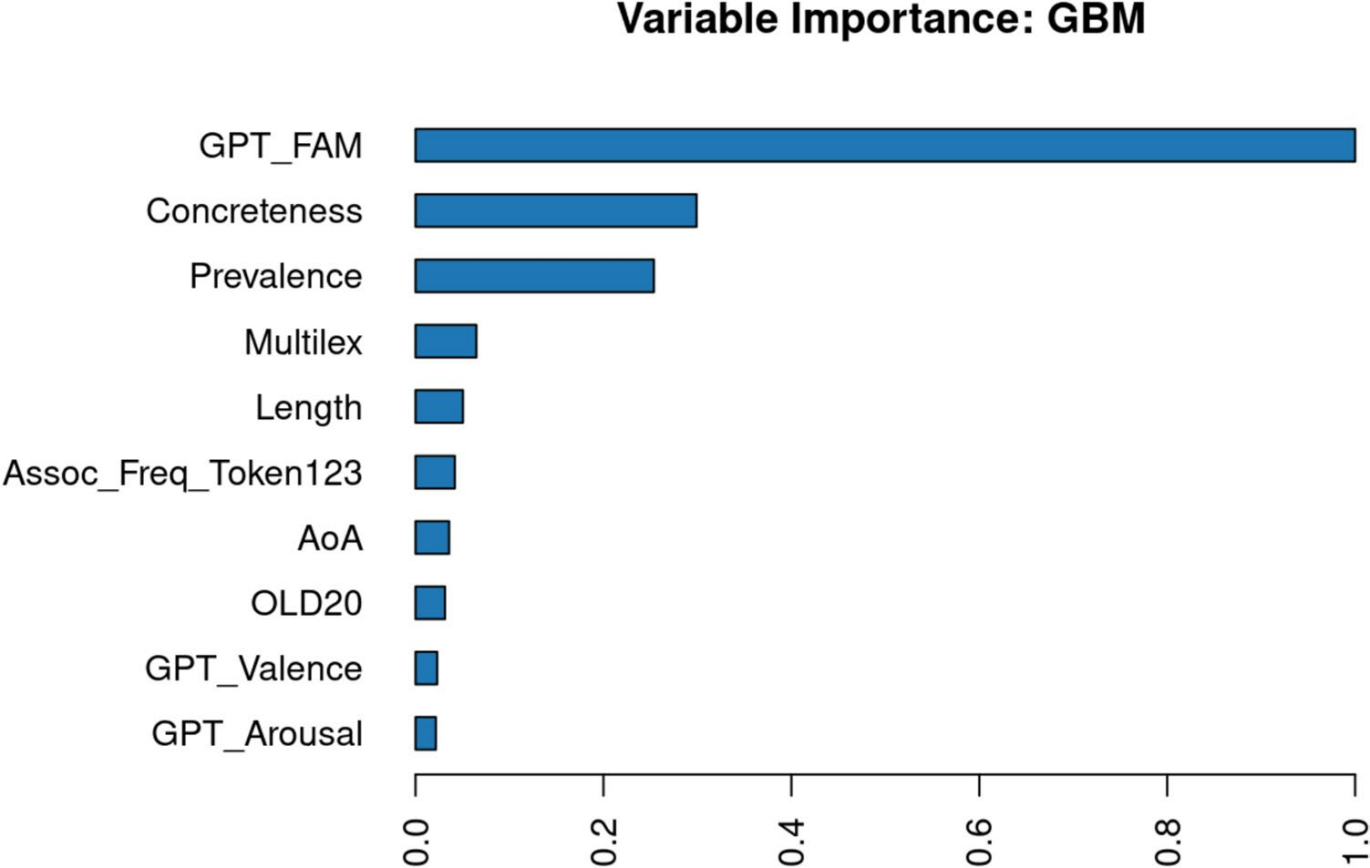
Importance of utility predictors as determined with the H2O AutoML package of Lee and Gates (2025)

The optimal model obtained *R*^2^ =.653 in a fivefold cross-validation on both the training data and the 25% left-out test data. Figure 4 shows the relative importance of the predictors. The most important predictor is GPT-generated word familiarity, followed by concreteness and word prevalence. Word frequency only comes in fourth place, followed by length. This suggests that the judged importance of a word is based primarily on how well known it is and its abstractness.

It is also revealing to look at the words that deviated from predicted utility on the basis of the lexical predictors. Table 2 shows the words which had much higher expected utility than predicted. A surprising number of these relate to health (e.g., “abscess,” “fibula,” “heartbeat”) or high-stakes situations (e.g., “bushfire,” “exam”). This may suggest that there is also a semantic or thematic dimension to expected utility, over and above the lexical dimensions we have analyzed so far.

**Table 2:** Words with the highest positive residuals from a linear regression analysis predicting utility

Word	Utility	Predicted Utility
abscess	0.57	0.33
confidant	0.71	0.49
washout	0.65	0.44
operable	0.68	0.46
biologic	0.57	0.36
debit	0.69	0.50
fibula	0.50	0.31
heartbeat	0.78	0.59
query	0.70	0.51
waken	0.63	0.44
protrude	0.60	0.42
atrophy	0.58	0.39
bushfire	0.53	0.35
telephone	0.79	0.61
exam	0.72	0.54
glassware	0.66	0.48
wellbeing	0.74	0.56
verbalize	0.69	0.51
blowup	0.55	0.38
jowl	0.46	0.28

**Fig. 5:**
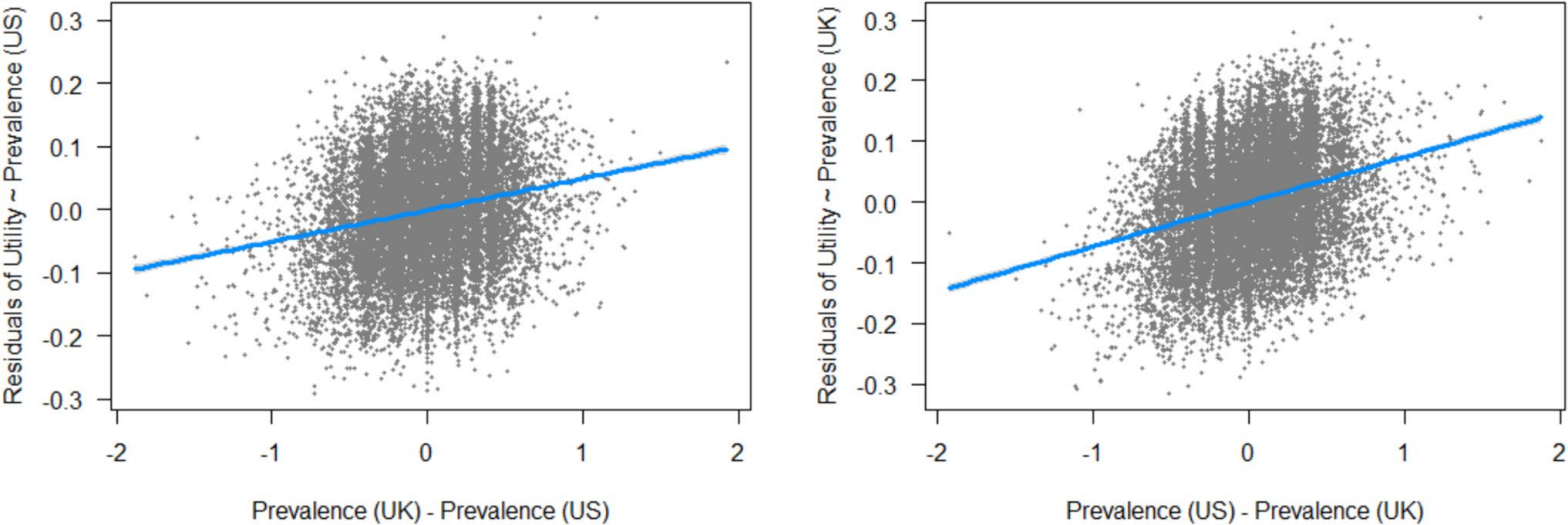
Regressions predicting the residuals of US word prevalence predicting utility from the difference between UK and US prevalence (left), and the residuals of UK word prevalence predicting utility from the difference between US and UK prevalence (right)

As reported in the Method section, we had a mix of US and UK participants in our sample; one may therefore wonder whether this had an effect on our utility ratings. We do not have enough observations to make a reliable distinction between US and UK utility, but since Brysbaert et al. (2016b) also provide prevalence norms for US and UK English separately, we can investigate the influence of relative differences between US and UK word knowledge. Figure 5 shows the outcome of regression models predicting the residuals when US prevalence predicts utility from the difference between UK and US prevalence, showing that the extent to which a word is more well known in the UK compared to the USA predicts a word's utility over and above US word knowledge alone (and vice versa). Words that have higher than expected utility based on US prevalence due to relatively higher UK word knowledge include “newsagent,” “rucksack,” “solicitor,” “austerity,” and “valuation.” Similarly, words like “chili,” “crosswalk,” “biracial,” “hibachi,” “cookout,” and “bipartisan” are more important than expected because they are relatively well known in US English. Thus, utility can be influenced by differences in regional varieties of English. However, when overall word prevalence (as used in the original analyses) is used to predict utility, the difference between US and UK prevalence is not a significant predictor over and above it (*t* =  − 1.62, *p* =.11), indicating that regional variation may be sufficiently captured in these measures and that our utility ratings reflect a good mix of both US and UK English.

**Fig. 6:**
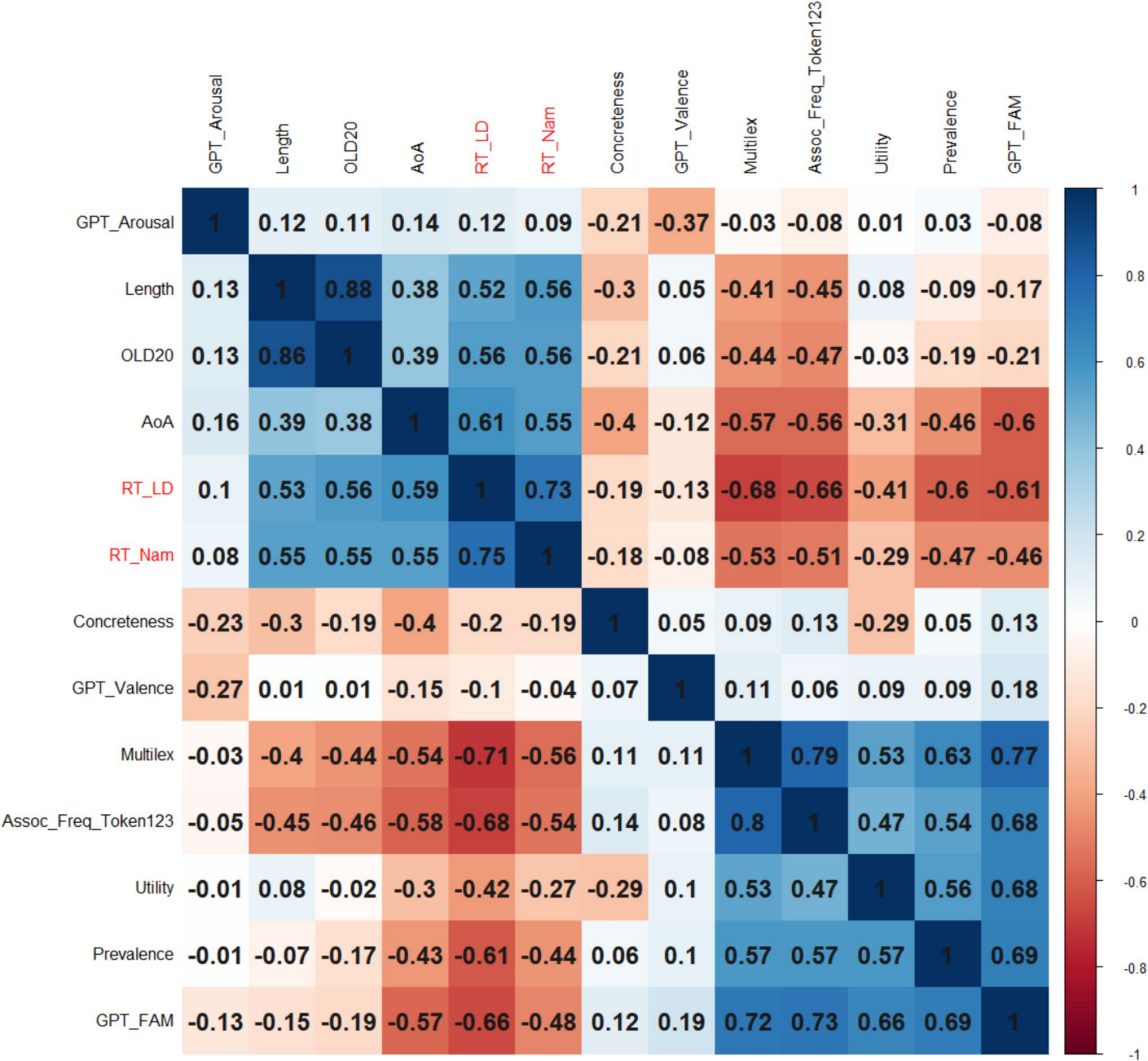
Correlations of word variables with response times in the English Lexicon Project: RT_LD = response time in lexical decision, RT_Nam = word naming latencies. Variables ordered with hierarchical clustering (which tends to put negatively correlated variables in separate clusters). Above the diagonal: Spearman correlations; below the diagonal: Pearson correlations

More interesting for practical purposes is to know how much expected utility contributes to explaining word processing times. An often-used database is the English Lexicon Project (Balota et al., 2007), containing lexical decision times and naming times for over 40,000 words. There were 15,704 content words for which all information was available. Figure 6 shows the correlations between the response times (standardized *z*-values) and the predictors.

In a linear regression analysis, all word variables except for concreteness (*p* =.40) were statistically significant predictors of lexical decision time, together accounting for 67.7% of the variance. The weakest significant predictor was GPT_Arousal (*p* =.003). Words with higher utility had faster lexical decision times (*t* =  − 9.89). For naming time, GPT_Valence and GPT_Arousal were nonsignificant predictors. Utility again negatively predicted naming time (*t* =  − 6.43), and the variables together accounted for 53.4% of the variance. Nonlinear multiple regressions with restricted cubic splines (three knots; Harrell, 2024) showed that none of the effects appeared to be misjudged because of nonlinear relationships.

**Fig. 7:**
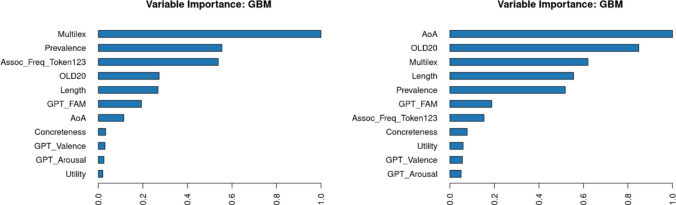
Importance of various variables in predicting response times in the English Lexicon Project. Left: lexical decision; right: word naming

Two new random forest analyses were conducted to determine the relative importance of the correlated predictors for lexical decision time and naming time. Figure 7 shows the outcome. Contrary to expectations, utility did not seem to explain much about differences in response times in addition to the other variables, either in lexical decision or in naming.

Another interesting database is the English Crowdsourcing Project (Mandera et al., 2020). This was an online vocabulary test in which participants indicated which words they knew in a list of words and nonwords. Its advantage is that the test was completed by a much more varied group of participants than the university students of the English Lexicon Project. For this database, word prevalence cannot be used, as the prevalence measure is based on the accuracy data from this study (Brysbaert et al., 2019b).

**Fig. 8:**
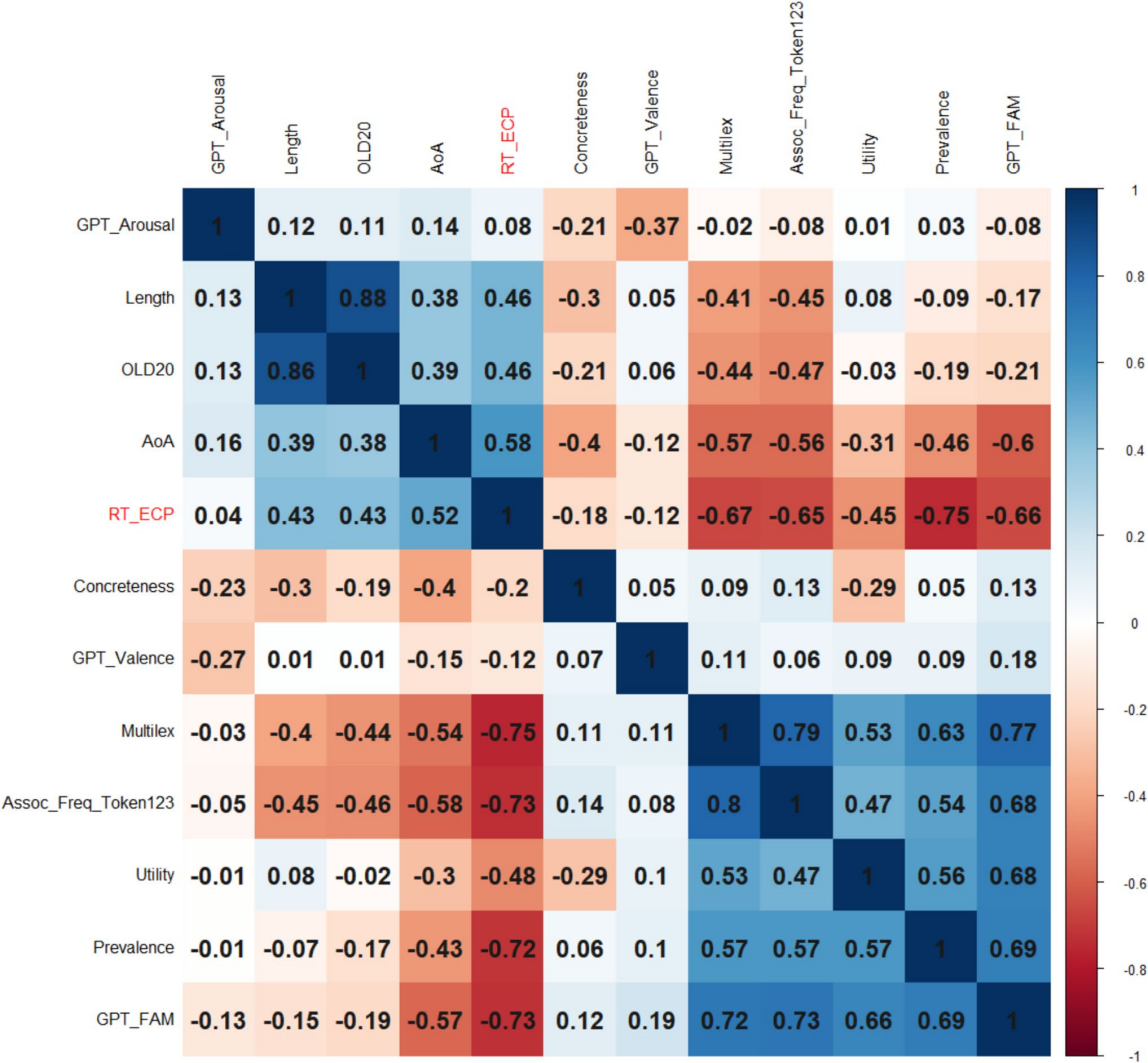
Correlations of word variables with response times in the English Crowdsourcing Project (RT_ECP). Variables ordered with hierarchical clustering (which tends to put negatively correlated variables in separate clusters). Above the diagonal: Spearman correlations; below the diagonal: Pearson correlations

Figure 8 shows the correlations between variables for the 15,704 words for which we had complete data.

**Fig. 9:**
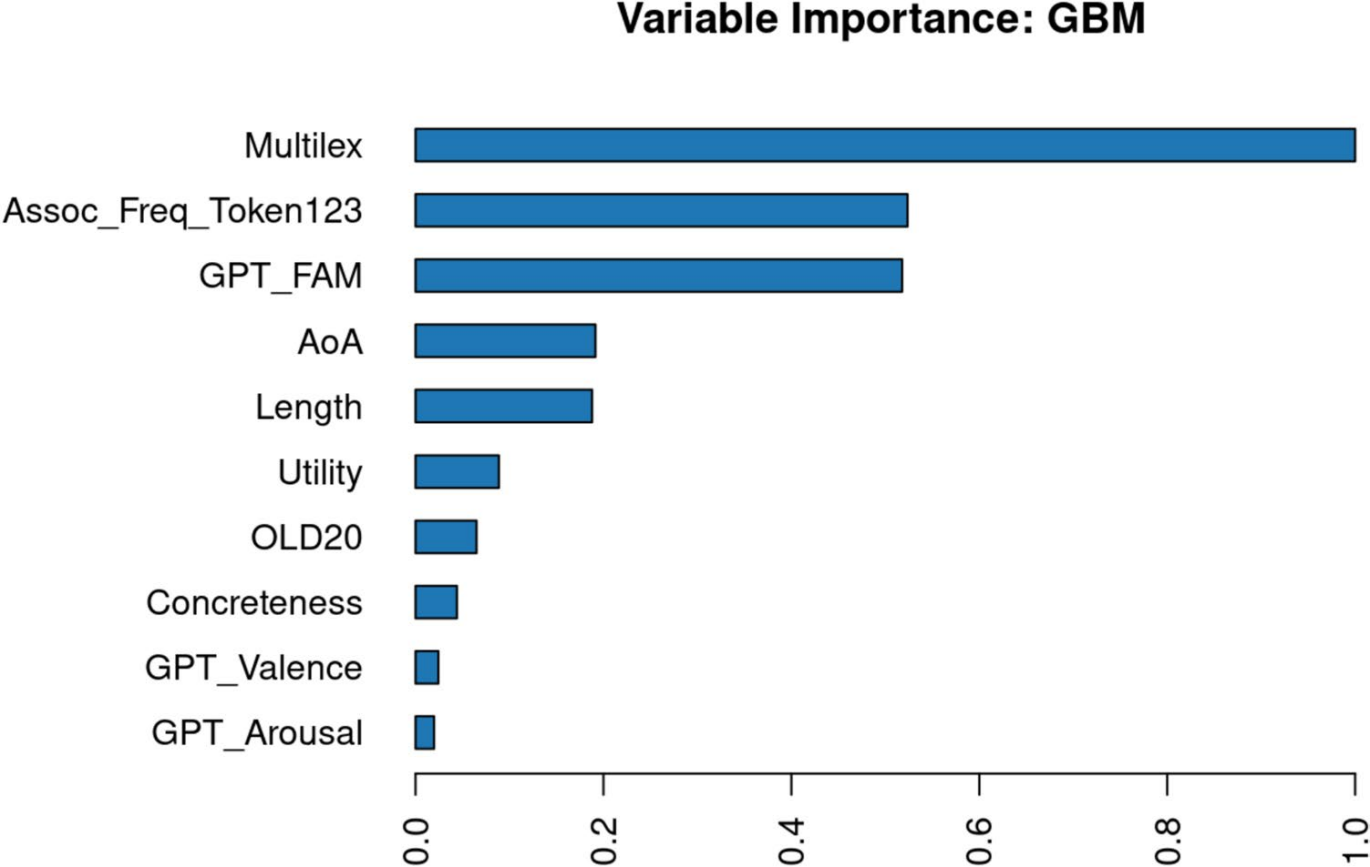
Relative importance of word variables in predicting response times in the English Crowdsourcing Project

A linear regression analysis showed that all predictors except AoA (*p* =.81) significantly predicted response times in the English Crowdsourcing Project, together accounting for 58.7% of the variance. Higher utility again predicted faster response times (*t* =  − 18.73). A random forest analysis (Lee & Gates, 2025) indicated that expected utility was only the fifth predictor in terms of importance, well after word frequency, semantic centrality, estimated familiarity, and word length (Fig. 9). In this analysis, word prevalence was not included, because it had been calculated based on the accuracy scores of the same study.

**Fig. 10:**
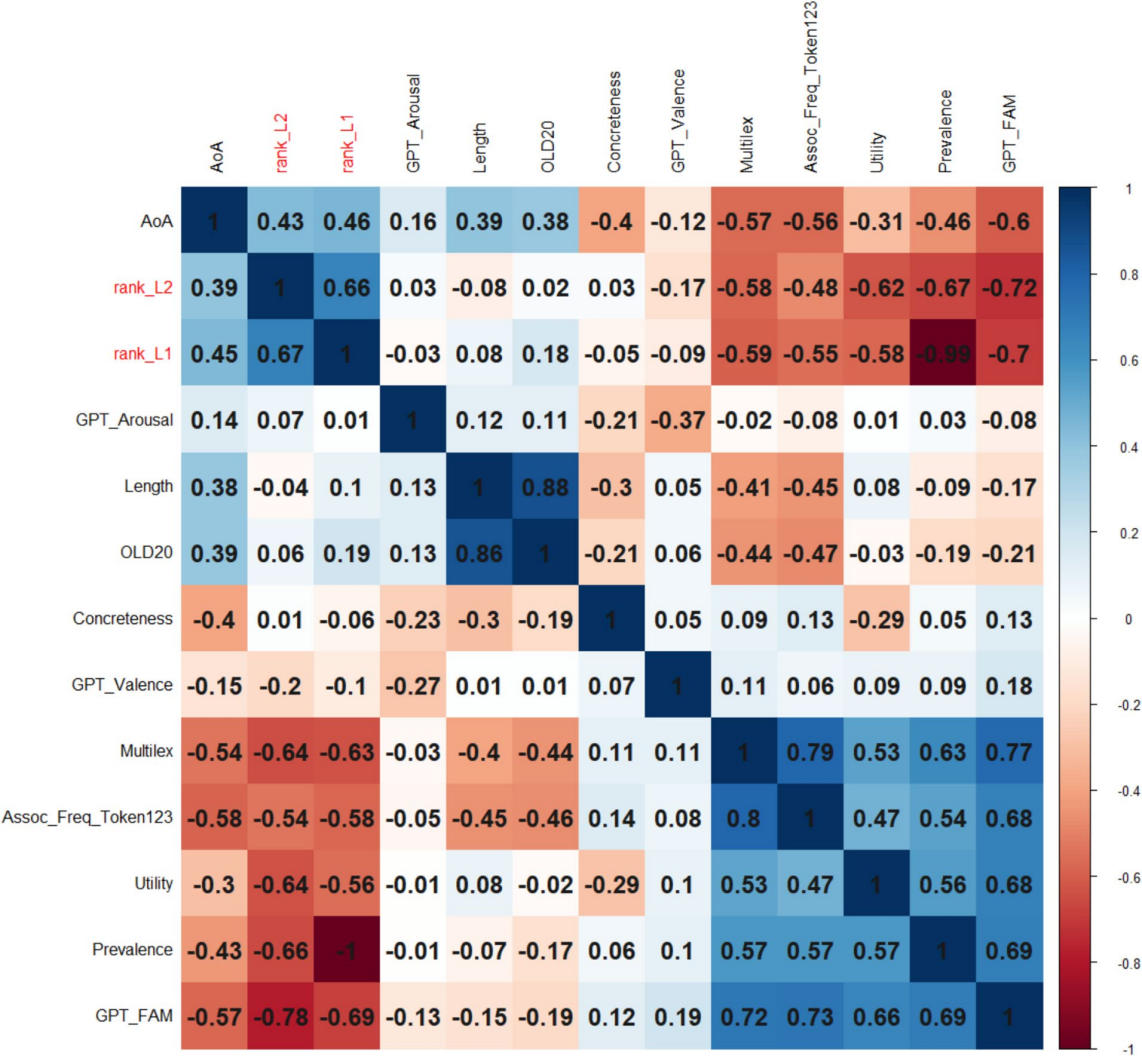
Correlations of word variables with word ranks in the English Crowdsourcing Project (rank_L2 and rank_L1). Variables ordered with hierarchical clustering (which tends to put negatively correlated variables in separate clusters). Above the diagonal: Spearman correlations; below the diagonal: Pearson correlations

The English Crowdsourcing Project also attracted many responses from second language (L2) speakers of English. These were analyzed by Brysbaert et al. (2021), who made a ranking of the most important words for L1 and L2 speakers, based on the percentage of speakers who knew the word and the time they needed to decide that the stimulus was a word. Figure 10 shows the correlations.

**Fig. 11:**
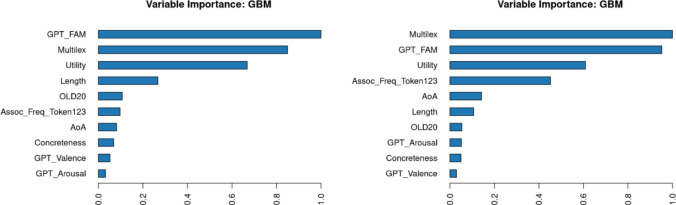
Importance of variables in predicting word knowledge in L2 speakers (left) and L1 speakers (right)

A random forest analysis (without word prevalence) indicated that expected utility was among the highest predictors of word rank (Fig. 11). For L2 speakers, words were more highly ranked when they were more familiar, were frequently observed in subtitles, had higher expected utility, and were longer. For L1 speakers, word length was replaced by semantic centrality, which may reflect the fact that L1 speakers have a more established semantic structure that is shared across speakers. Note that the word ranks were mainly determined by accuracy (i.e., word knowledge). Overall, it appears that utility is a better variable for predicting which words will be known than for predicting how fast they will be recognized.

**Fig. 12:**
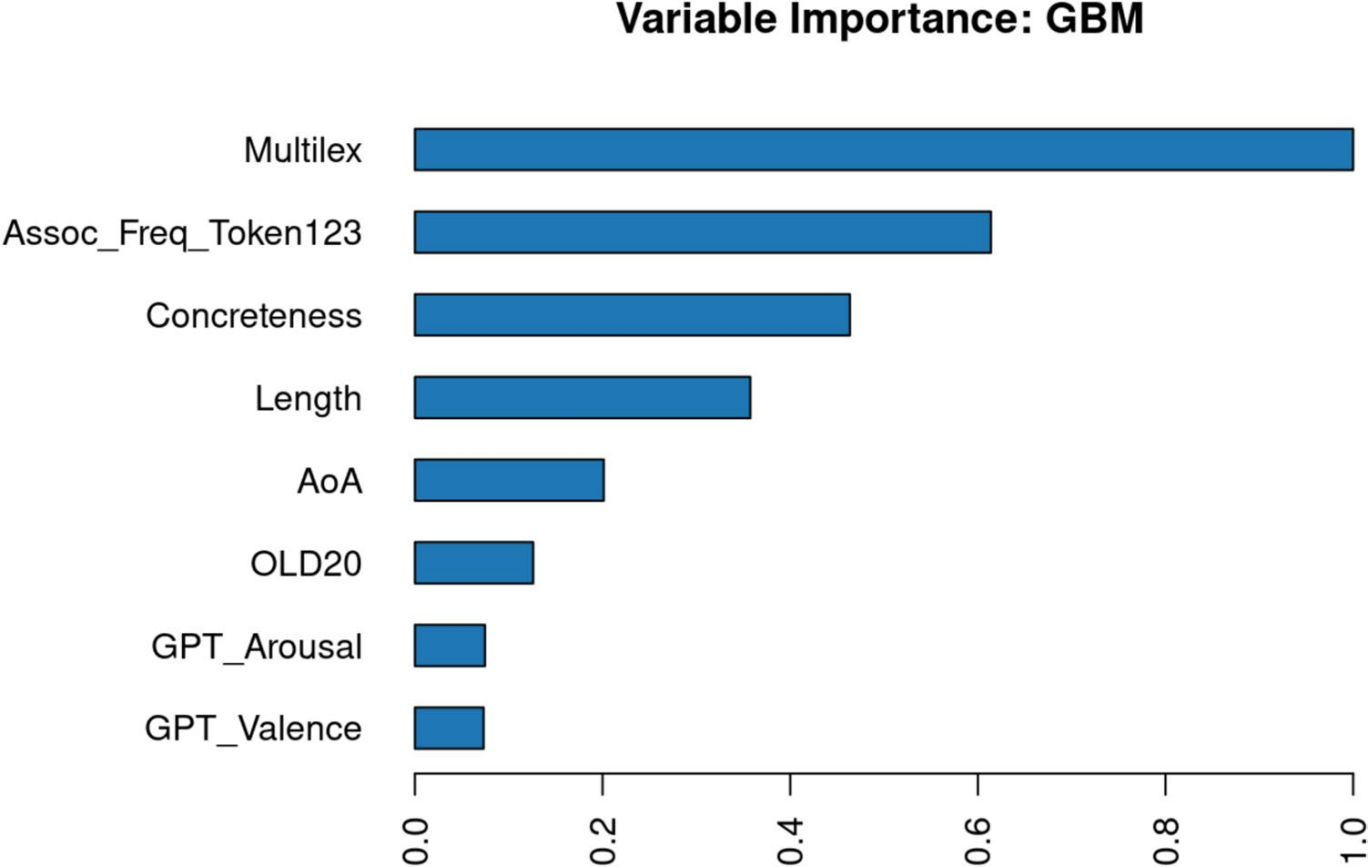
Importance of variables in predicting utility when GPT familiarity and prevalence are excluded as predictors

The finding that expected utility is chiefly important for predicting word knowledge is consistent with the intuition noted in the introduction that people may be more likely to learn and retain certain words because they perceive them as more useful. It also relates to the concept of core words, in which the most important words in language may be captured by their lexical characteristics. It may therefore be interesting to explore what factors predict expected utility leaving aside any factors related to word knowledge, under the assumption that the importance of words goes on to influence how well they are known. Figure 12 shows the most important predictors of expected utility if GPT familiarity and prevalence, which both reflect word knowledge, are excluded from the model. The most important variables for predicting expected utility are then frequency, semantic centrality, and concreteness (with more abstract words again being seen as more useful).

### Analysis of multiword expressions

**Fig. 13:**
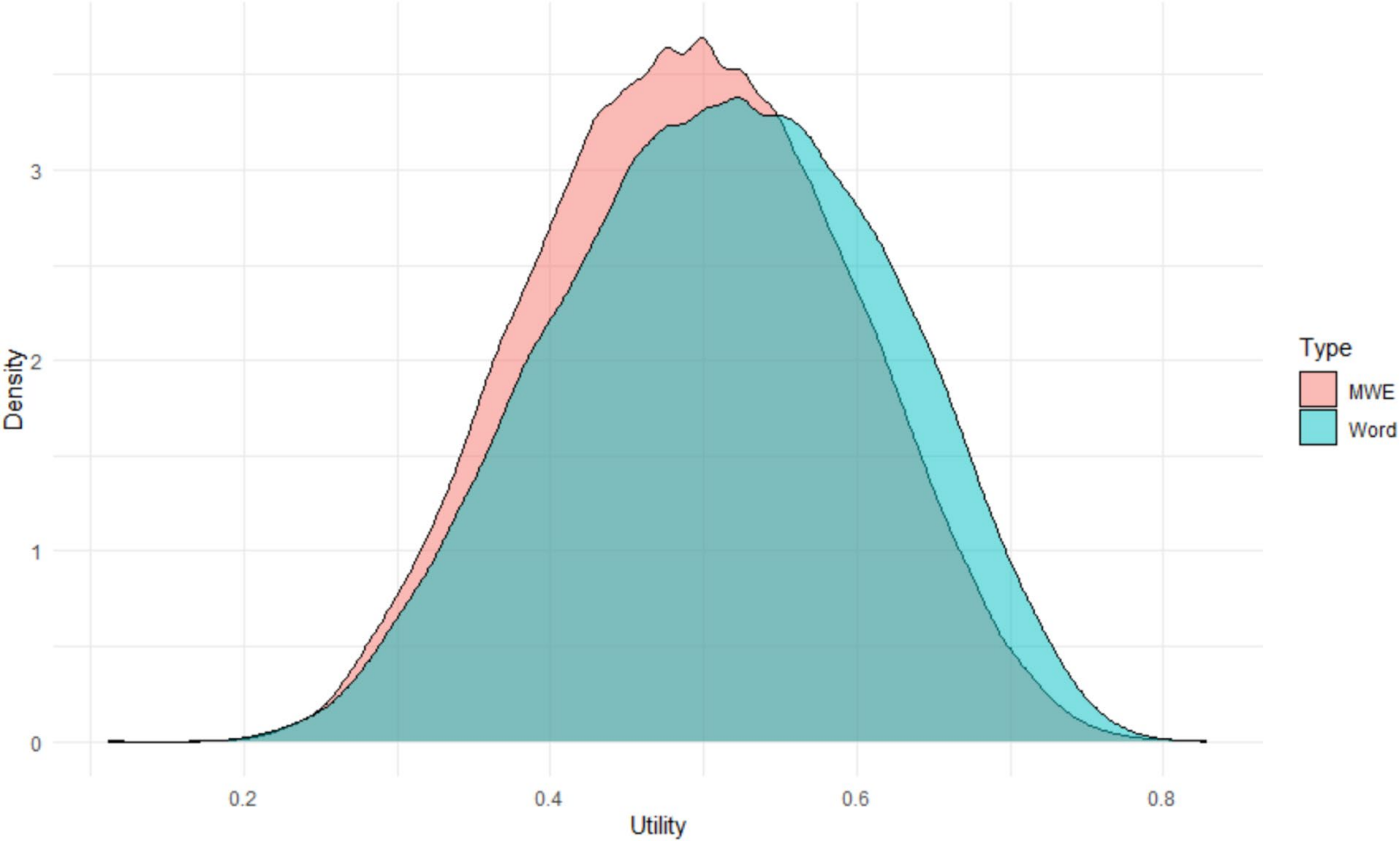
Probability distributions of utility scores for words and multiword expressions (MWE). Graph made with ggplot2 (Wickham, 2011)

Figure 13 shows the expected utility scores of multiword expressions (MWE) compared to words. Although words in general are perceived as more useful than MWEs, there is a large overlap of distributions. Indeed, every second language learner will tell you that some MWEs are among the first stimuli learned (e.g., good morning, thank you). Table 3 includes the 20 MWEs with the highest expected utility and the 20 MWEs with the lowest expected utility. Two of the three expressions with the lowest expected utility were used as control items.

**Table 3:** Expressions with the highest expected utility scores (left) and the lowest expected utility (right)

Expression	Utility	Expression	Utility
help me	0.83	how now brown cow	0.17
thank you very much	0.81	crissal thrasher	0.18
no thank you	0.80	savo finnish	0.18
what's going on	0.80	benzene ring	0.18
I love you	0.80	Raggedy Ann	0.19
pay attention	0.80	eurasian badger	0.19
you're welcome	0.80	tamil tigers	0.19
ask a question	0.80	walrus moustache	0.20
not yet	0.80	muzzle velocity	0.20
final decision	0.80	morel mushrooms	0.20
I miss you	0.80	kelp bed	0.20
be careful	0.80	anzac day	0.20
my name is	0.79	tramp stamp	0.20
thank you so much	0.79	ghetto fabulous	0.20
I am	0.79	turtle soup	0.21
how much does it cost	0.79	k mart	0.21
talk about	0.78	moon boot	0.21
I need your help	0.78	gaussian curve	0.21
I have a question	0.78	jollof rice	0.21
go to the bathroom	0.78	Dorian Gray	0.21

**Fig. 14:**
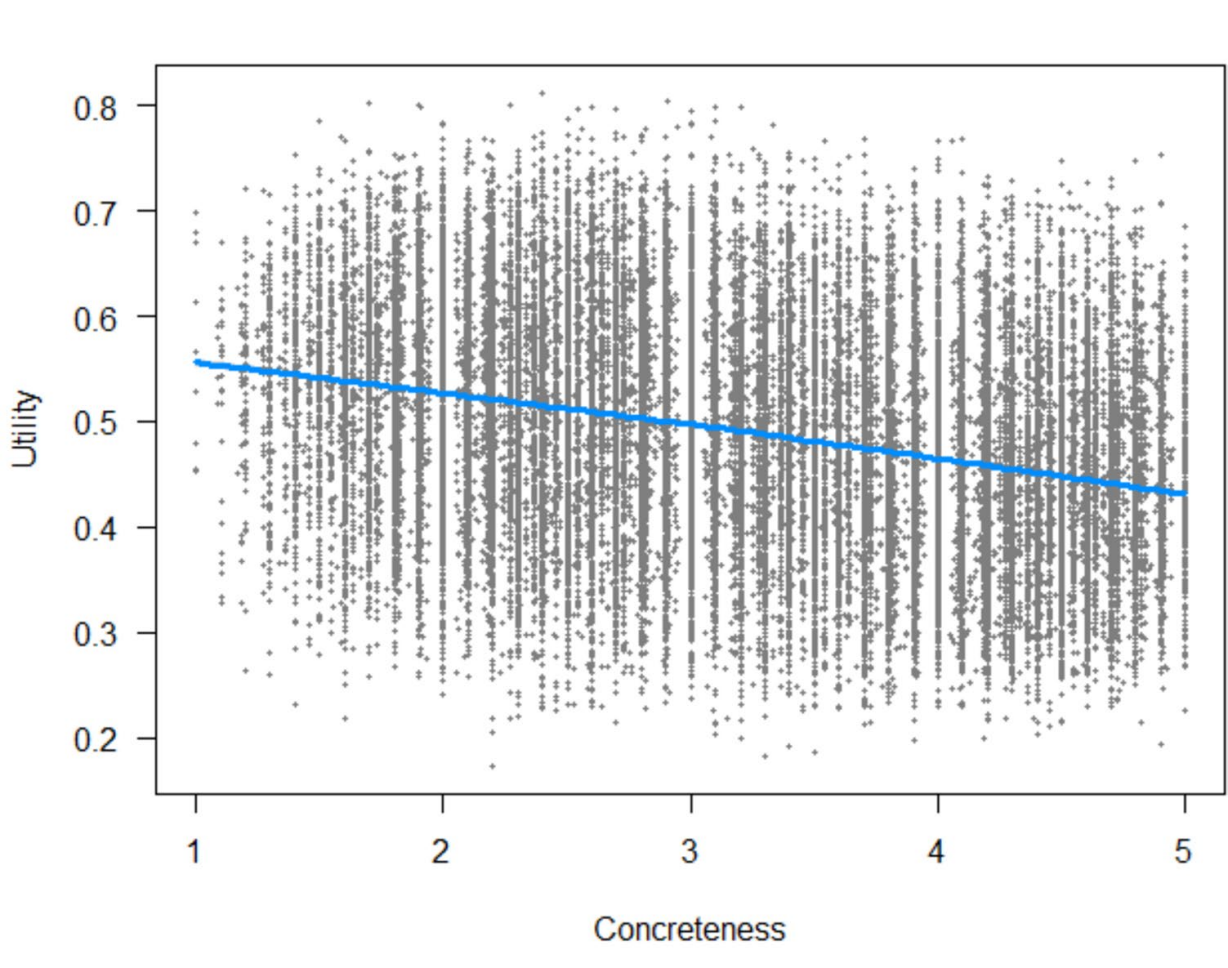
Scatterplot of the negative correlation between MWE concreteness and expected utility. The blue line is the regression line from a nonlinear restricted cubic spline regression analysis, showing that the relationship is effectively linear (figure created with the visreg and rms R packages; Harrell, 2024)

So far, we have only one set of human ratings for MWEs that we can use as a predictor. It is the concreteness ratings collected by Muraki et al. (2023). As we observed for words, the correlation between expected utility and concreteness is negative and substantial (*r* =  −.31, *N* = 42,970). Figure 14 shows the scatterplot.

**Fig. 15:**
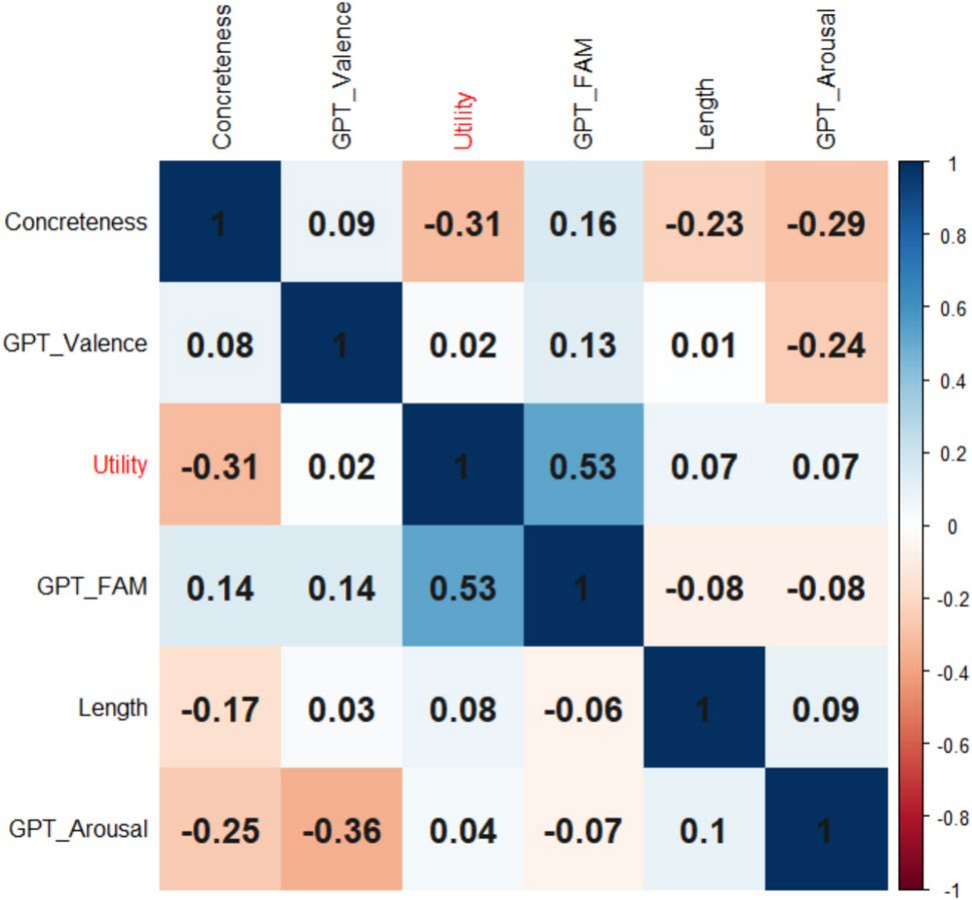
Correlations of expected utility scores with other features of MWEs. Above the diagonal: Spearman correlations; below the diagonal: Pearson correlations

Turning to AI estimates, Martínez et al. (2025) obtained GPT-4o estimates of valence and arousal for MWEs, and Brysbaert et al. (2025) collected GPT-4o estimates of familiarity. Figure 15 shows the correlations. Apart from the negative correlation with concreteness already reported, there is a large positive correlation with GPT-estimated familiarity, as could be foreseen. Expected utility did not differ much as a function of MWE length, estimated valence, or estimated arousal, in contrast to words where longer words have higher expected utility.

**Fig. 16:**
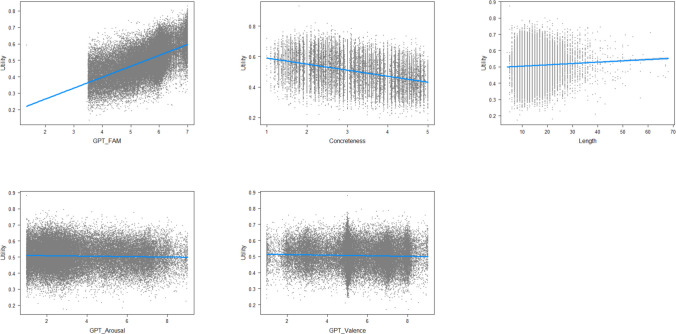
Scatterplots of unique effects of the predictors in a regression analysis in predicting the expected utility of MWEs. Note the few stimuli with low GPT fam values. These were part of the control items

**Fig. 17:**
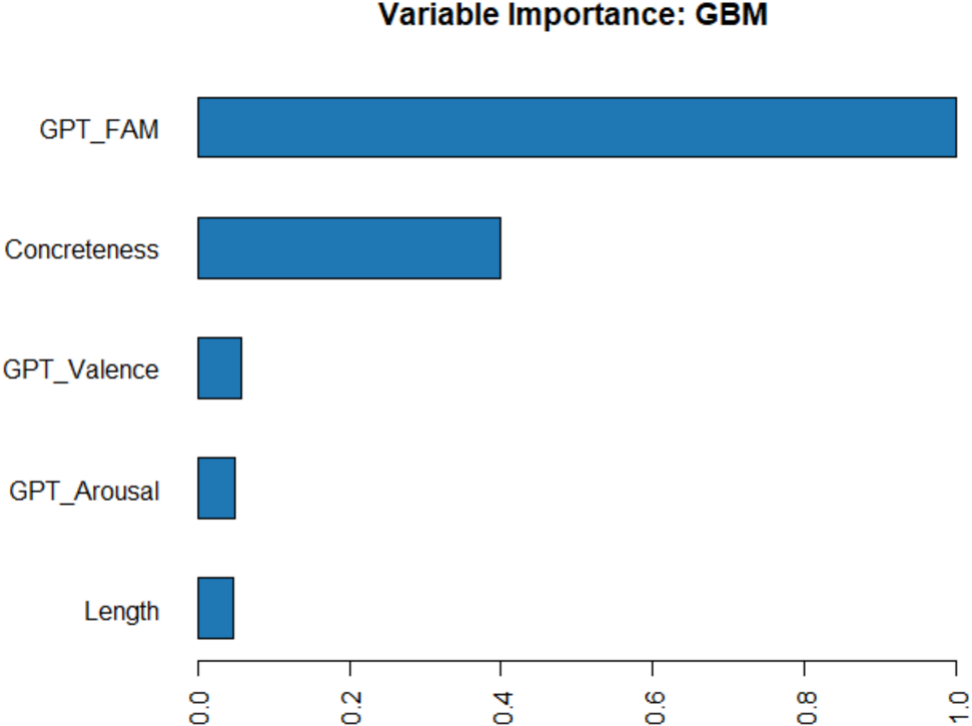
Relative importance of the predictors in a regression analysis in predicting the expected utility of MWEs according to a random forest analysis (Lee & Gates, 2025)

Figures 16 and 17 show the relative importance of variables for predicting the expected utility of MWEs, confirming that GPT familiarity and concreteness are by far the most important ones.

The effects of the predictors are mostly in line with the results for words. GPT familiarity and concreteness are strong positive and negative predictors of expected utility, respectively. However, a very different result is observed for length. In contrast to the strong positive effect for words, expected utility differs very little by MWE length. It has dropped down to the least important predictor of utility, after estimated valence and arousal.

## Discussion

This article examines the extent to which verbal processing depends on the utility that speakers expect to derive from knowing a word or expression. Until now, vocabulary acquisition has been considered comparable to computer learning, in which only stimulus-related factors such as frequency of occurrence and convergence/divergence in form-meaning mappings are important. This contradicts the impression that most people (including the authors) have that some words are easier to acquire than others, even though there are no language-related differences between them.

People are constantly confronted with more stimuli than they can process and store in their memory. As a result, they are selective in what they process and remember, a process called selective attention (James, 1890; Johnston & Dark, 1986; Van Ede & Nobre, 2023; Wundt, 1880). A similar process seems likely to occur with verbal knowledge. The number of word forms that people are confronted with is almost limitless, as evidenced by the fact that the number of word types continues to increase at a constant rate even in corpora of trillions of words (Brysbaert et al., 2016a). Most new word types are not worth remembering (e.g., because they are typos or speech errors), only about 50,000 words are commonly shared, and even fewer in less educated groups.^4^ The question is whether we can operationalize the perceived usefulness of word knowledge.

We made an initial attempt by explicitly asking people about the expected usefulness of knowing specific words and expressions. We could have done this by asking people to rate words and expressions on a Likert scale, but we opted for the best–worst technique (Hollis, 2018, 2020). The main reason for this was that the best–worst technique seems to yield better results for less familiar characteristics (Heo et al., 2022; Hollis & Westbury, 2018; Mohammad, 2018). We also expected data collection to be more efficient, but this turned out not to be the case. We needed 5,500 participants, each working for 12 min. To have 12 participants rate the 82,000 stimuli with a 10% overlap in the control stimuli, we would have needed 1,000 participants, each rating 1,000 stimuli (which takes about an hour).

The findings showed a high level of agreement regarding the usefulness of words and expressions, indicating that it was a meaningful variable for the participants. The findings were also largely in line with expectations. Stimuli were rated as more useful if they were more frequent, widely known, learned early in life, and central to the semantic network. Word knowledge (i.e., GPT familiarity and prevalence) was the best variable to predict utility, which may have arisen partly because participants were explicitly instructed to rate unknown words as “least useful,” but the strong relationship between utility and word knowledge for highly familiar stimuli suggests that this is unlikely to be the full explanation. Negative words were also rated as slightly more useful than positive ones, in line with a general negativity bias in language (Bebbington et al., 2017; Rozin et al., 2010; Rozin & Royzman, 2001; but see Dodds et al., 2015). Our findings further showed that the usefulness of words was influenced by differences in US and UK English, but also that our ratings overall seemed to capture a good mix of this regional variation.

In addition, longer words were judged as more useful, all other factors being equal. This is a somewhat surprising finding, standing in contrast to the well-established advantage for shorter words in visual word processing (Barton et al., 2014), short-term memory (Baddeley et al., 1975), and the statistics of language use (Piantadosi et al., 2011; Zipf, 1936). It is possible that longer words are seen as a signal of importance, since they encode more conceptually complex meanings (Lewis & Frank, 2016), which people may think are useful to know. Longer words are also richer in information content (Piantadosi et al., 2011), so while longer words may not have a processing advantage, they are often highly informative and convey information not provided by the context, and may therefore be seen as more useful to know. Notably, the effect of length was much less important for MWEs than for words.

At the same time, the results were slightly less satisfactory than hoped for. The main reason for this is that the recently introduced GPT familiarity estimates (Brysbaert et al., 2025) largely overlap with the expected utility scores (total correlation, *r* =.58). Not only is it the best predictor of expected utility (Fig. 4), but in several analyses it also largely eliminated the impact that expected utility would have (see Fig. 11 in particular). Much depends here on the theoretical importance attached to AI-generated familiarity estimates. These are based on an assessment of word usage in a very large corpus (1,000 times larger than individuals can master) and depend on the way texts are split into tokens and processed by a multilayer transformer network. They correlate strongly with word knowledge, but mainly because they reflect shared underlying processes. The same applies to estimates of word prevalence. These also correlate strongly with word knowledge, but again do not provide much information about the underlying causes. In this respect, expected usefulness may be a more fundamental factor, as it explains why some words are more likely to be known than others (via the underlying attention mechanism; Brod, 2021).

Of course, the next question is why some words are perceived as more useful or important to know than others. One answer is that it relates to the individual needs and personal values of the user, such as the finding that people are more likely to remember information that they have just failed to answer a question about (Brod, 2021; Kornell, 2014; Pan & Carpenter, 2023). It seems likely that words that relate to an individual’s own experience and circumstances would be perceived as more useful to them.

Another answer lies in the idea that the core words in language can be captured by the lexical properties of words, such as frequency or semantic centrality. Indeed, if the variables which reflect word knowledge are left out of the equation, it appears that frequency, semantic centrality, and concreteness capture a large part of the importance of words. This in turn may influence how likely they are to be learned and known by a speaker.

However, our predictors do not capture all of the variance in expected utility; only around two thirds of the systematic variance is accounted for by the predictors we included in Figs. 3 and 4. It is therefore also interesting to look at the deviations of expected utility from the predictions, which Table 2 gives some indication of, to understand what makes words important apart from those variables. One possibility is that there is also a semantic dimension to expected utility, where certain meanings are considered more useful or important to know. Looking further into the residuals of the words reveals some common themes. Some themes are consistently higher in utility than predicted, such as health (“operable,” “heartbeat,” “wellbeing”), money (“debit,” “payday,” “fee”), marriage (“annulment,” “spouse,” “fiancée”), food (“menu,” “food”), and weather (“rain,” “storm”). Similarly, others are lower in utility than predicted, such as childish words (“oink,” “sissy,” “moo,” “abracadabra,” “peekaboo”).

We made the assumption that expected utility should influence how well words are known. However, we also included analyses in which word knowledge (e.g., prevalence) predicts utility. This raises an important question: are words more useful because they are known by more people, or are words known by more people because they are useful? The answer is likely more complicated: word usefulness may influence how widely a word is known, which may then further increase its usefulness, and so on in a feedback loop. Speakers are of course attuned to what other speakers are doing, and if a word is widely known by many speakers, it may serve as a signal that that word is useful to know. This question of directionality extends to other factors as well. For example, the most frequent words may be the most important, or they may be the most frequent precisely because they are the most important (Stubbs, 1986). Indeed, the amount of available data on words nowadays is extensive, but researchers still do not have a clear picture about how all of these variables are related. This suggests a promising future direction in analyzing the relationships between lexical data on a large scale, to systematically understand the interrelationships between these different characteristics, the latent constructs they measure, and how these variables may influence each other. A structural equation model or exploratory graph analysis may provide a starting point for such an analysis. Some studies have given an initial direction for answering these questions (e.g., Gao et al., 2023), but much remains to be understood about how the extent of available lexical data can be integrated into a coherent framework. A concrete step towards this goal is being undertaken by the PsychLing-101 project (https://github.com/Data-X01/PsychLing-101), which seeks to compile an extensive database of psycholinguistic data and provides the possibility of using LLMs to explore and provide insights into the relationships between these measures.

Another notable finding is that expected utility has a stronger effect on word knowledge (accuracy) than on word recognition speed (measured with lexical decision and word naming). This may indicate that the online mechanisms of word recognition are primarily language-driven and insensitive to motivational factors. In this regard, Gao et al. (2022) reported an interesting difference between visual and auditory lexical decision. Negative words are answered faster in auditory lexical decisions than in visual lexical decisions, leading the authors to hypothesize that valence partially influences word recognition at the sensory-perceptual stage, due to the effects of positive and negative reinforcers on perception. It is possible that visual word processing is less influenced by everyday preoccupations than auditory word processing, because reading takes place in shielded spaces.

The most surprising finding was the significant negative correlation between concreteness and expected utility. Participants found abstract words more useful to know than concrete words. This contradicts the widespread assumption of a concreteness advantage: concrete words are assumed to be easier to process and remember than abstract words (Fliessbach et al., 2006; Jessen et al., 2000). Some questions about a general concreteness effect have come from large-scale mega-studies, in which the correlation between word processing performance and concreteness is usually absent or even negative when all words are taken into account (Brysbaert et al., 2016b, 2019a; Mandera et al., 2020).

One way to understand the different effects of word concreteness may be to distinguish between the concrete words we learned as children and concrete words that refer to things we rarely name or with which we have little direct experience. The first words a child learns are grounded in interactions with the world and are usually concrete. They form the building blocks on which later language development is based and, as such, probably have a processing advantage. However, the same does not apply to many other object names. We do not know the specific names of many things we use and refer to them as “that thing” or by pointing at them. Moreover, concrete concepts form hierarchies, in which general nouns can be used to refer to specific cases (animal, dog, terrier, Schipperke) and lower categories are named with compound words (turtle soup). If one knows the words “turtle” and “soup,” it is not necessary to also know the name turtle soup, unless the compound word “turtle soup” expresses more than the sum of turtle and soup (as is the case, for example, with snowman and basketball referring to the ball itself). Abstract words, on the other hand, often refer to feelings, rights, and duties, which are important to know in everyday life. Abstract words are also probably more useful for social interactions, for example, in talking about politics, work, or relationships. Words and expressions that serve pragmatic functions (e.g., “please” and “thank you”) also probably tend to be more abstract.

Another strength of the current manuscript is that it provides new people-based information for nearly 48,000 English multiword expressions. As mentioned above, there are only two sets of human ratings available for these stimuli: the concreteness ratings collected by Muraki et al. (2023) and the anxiety associations collected by Mohammad (2026). The current utility norms provide large-scale information about the usability of English expressions, which is important information in educational situations (for both first and second languages). The expected utility scores complement the GPT-generated familiarity estimates and are likely to be more in line with people's actual interests.

It will be interesting to see what other applications the expected utility scores yield. A pleasant aspect of making norms available is that they are often used in ways that no one could have foreseen.

## Data Availability

The expected utility ratings are available at https://osf.io/4nwgx/. There are files with the raw data, and there is an overall file for all 82,880 words and expressions. The OSF repository also contains the R code used in the analyses reported here.

## Footnotes

1 The authors thank Emmanuel Keuleers, who raised the question in informal talks around 2010.

2 Prolific could improve its quality control if researchers could easily indicate after a study which participants had to be excluded due to useless responses, without these participants being excluded from payment.

3 As $\mathrm{248,640}$ is not divisible by 45, a few items were presented more than 18 times.

4 Importantly, these estimates do not include multiword expressions, a gap in the existing literature.

## References

[CR1] Baddeley, A. D., Thomson, N., & Buchanan, M. (1975). Word length and the structure of short-term memory. *Journal of Verbal Learning and Verbal Behavior,**14*(6), 575–589.

[CR2] Balota, D. A., Yap, M. J., Hutchison, K. A., Cortese, M. J., Kessler, B., Loftis, B., Neely, J. H., Nelson, D. L., Simpson, G. B., & Treiman, R. (2007). The English Lexicon Project. *Behavior Research Methods,**39*(3), 445–459. 17958156 10.3758/bf03193014

[CR3] Barton, J. J. S., Hanif, H. M., Eklinder Björnström, L., & Hills, C. (2014). The word-length effect in reading: A review. *Cognitive Neuropsychology,**31*(5–6), 378–412. 24665973 10.1080/02643294.2014.895314

[CR4] Bebbington, K., MacLeod, C., Ellison, T. M., & Fay, N. (2017). The sky is falling: Evidence of a negativity bias in the social transmission of information. *Evolution and Human Behavior,**38*(1), 92–101.

[CR5] Bell, H. (2012). Core vocabulary. The Encyclopedia of Applied Linguistics.

[CR6] Breheny, P., & Burchett, W. (2017). Visualization of regression models using visreg. *The R Journal,**9*, 56–71.

[CR7] Brod, G. (2021). Predicting as a learning strategy. *Psychonomic Bulletin & Review,**28*(6), 1839–1847. 33768503 10.3758/s13423-021-01904-1 PMC8642250

[CR8] Brysbaert, M., & Drieghe, D. (2003). Please stop using word frequency data that are likely to be word length effects in disguise. *Behavioral and Brain Sciences,**26*(4), 479–479.

[CR9] Brysbaert, M., Keuleers, E., & Mandera, P. (2019a). Recognition times for 54 thousand Dutch words: Data from the Dutch Crowdsourcing Project. *Psychologica Belgica,**59*(1), 281. 31367458 10.5334/pb.491 PMC6659767

[CR10] Brysbaert, M., Keuleers, E., & Mandera, P. (2021). Which words do English non-native speakers know? New supernational levels based on yes/no decision. *Second Language Research,**37*(2), 207–231.

[CR11] Brysbaert, M., Mandera, P., & Keuleers, E. (2018). The word frequency effect in word processing: An updated review. *Current Directions in Psychological Science,**27*(1), 45–50.

[CR12] Brysbaert, M., Mandera, P., McCormick, S. F., & Keuleers, E. (2019b). Word prevalence norms for 62,000 English lemmas. *Behavior Research Methods,**51*, 467–479. 29967979 10.3758/s13428-018-1077-9

[CR13] Brysbaert, M., Martínez, G., & Reviriego, P. (2025). Moving beyond word frequency based on tally counting: AI-generated familiarity estimates of words and phrases are an interesting additional index of language knowledge. *Behavior Research Methods,**57*(28), 1–15. 10.3758/s13428-024-02561-7 39733132

[CR14] Brysbaert, M., New, B., & Keuleers, E. (2012). Adding part-of-speech information to the SUBTLEX-US word frequencies. *Behavior Research Methods,**44*(4), 991–997. 22396136 10.3758/s13428-012-0190-4

[CR15] Brysbaert, M., Stevens, M., Mandera, P., & Keuleers, E. (2016a). How many words do we know? Practical estimates of vocabulary size dependent on word definition, the degree of language input and the participant’s age. *Frontiers in Psychology,**7*, 1116. 27524974 10.3389/fpsyg.2016.01116 PMC4965448

[CR16] Brysbaert, M., Stevens, M., Mandera, P., & Keuleers, E. (2016b). The impact of word prevalence on lexical decision times: Evidence from the Dutch Lexicon Project 2. *Journal of Experimental Psychology: Human Perception and Performance,**42*(3), 441. 26501839 10.1037/xhp0000159

[CR17] Brysbaert, M., Warriner, A. B., & Kuperman, V. (2014). Concreteness ratings for 40 thousand generally known English word lemmas. *Behavior Research Methods,**46*(3), 904–911. 24142837 10.3758/s13428-013-0403-5

[CR18] Calude, A. S., & Pagel, M. (2011). How do we use language? Shared patterns in the frequency of word use across 17 world languages. *Philosophical Transactions of the Royal Society b: Biological Sciences,**366*(1567), 1101–1107. 10.1098/rstb.2010.0315 PMC3049087 21357232

[CR19] Calude, A. S., & Pagel, M. (2014). Frequency of use and basic vocabulary. In Multilingual Cognition and Language Use: Processing and Typological Perspectives (pp. 45–72). John Benjamins Publishing Company.

[CR20] Carter, R. (1998). Vocabulary: Applied linguistic perspectives. Routledge.

[CR21] Chang, M., Jones, M. N., & Johns, B. T. (2023). Comparing word frequency, semantic diversity, and semantic distinctiveness in lexical organization. *Journal of Experimental Psychology: General,**152*(6), 1814–1823. 37307352 10.1037/xge0001407

[CR22] Cisek, P. (1999). Beyond the computer metaphor: Behaviour as interaction. *Journal of Consciousness Studies,**6*(11–12), 125–142.

[CR23] De Deyne, S., Navarro, D. J., Perfors, A., Brysbaert, M., & Storms, G. (2019). The “small world of words” English word association norms for over 12,000 cue words. *Behavior Research Methods,**51*(3), 987–1006. 30298265 10.3758/s13428-018-1115-7

[CR24] de Leeuw, J. R., Gilbert, R. A., & Luchterhandt, B. (2023). jsPsych: Enabling an open-source collaborative ecosystem of behavioral experiments. *Journal of Open Source Software,**8*(85), Article 5351.

[CR25] Dodds, P. S., Clark, E. M., Desu, S., Frank, M. R., Reagan, A. J., Williams, J. R., Mitchell, L., Harris, K. D., Kloumann, I. M., Bagrow, J. P., Megerdoomian, K., McMahon, M. T., Tivnan, B. F., & Danforth, C. M. (2015). Human language reveals a universal positivity bias. *Proceedings of the National Academy of Sciences,**112*(8), 2389–2394. 10.1073/pnas.1411678112 PMC4345622 25675475

[CR26] Ellis, A. W., & Ralph, L. M. A. (2000). Age of acquisition effects in adult lexical processing reflect loss of plasticity in maturing systems: Insights from connectionist networks. *Journal of Experimental Psychology: Learning, Memory, and Cognition,**26*(5), 1103–1123. 10.1037/0278-7393.26.5.1103 11009247 10.1037//0278-7393.26.5.1103

[CR27] Fishburn, P. C. (1981). Subjective expected utility: A review of normative theories. *Theory and Decision,**13*(2), 139–199.

[CR28] Fliessbach, K., Weis, S., Klaver, P., Elger, C. E., & Weber, B. (2006). The effect of word concreteness on recognition memory. *NeuroImage,**32*(3), 1413–1421. 16861011 10.1016/j.neuroimage.2006.06.007

[CR29] Gao, C., Shinkareva, S. V., & Desai, R. H. (2023). Scope: The South Carolina psycholinguistic metabase. *Behavior Research Methods,**55*(6), 2853–2884. 35971041 10.3758/s13428-022-01934-0 PMC10231664

[CR30] Gao, C., Shinkareva, S. V., & Peelen, M. V. (2022). Affective valence of words differentially affects visual and auditory word recognition. *Journal of Experimental Psychology: General,**151*(9), 2144–2159. 10.1037/xge0001176 35113643 10.1037/xge0001176 PMC7616442

[CR31] Grainger, J., & Jacobs, A. M. (1996). Orthographic processing in visual word recognition: A multiple read-out model. *Psychological Review,**103*(3), 518–565. 10.1037/0033-295X.103.3.518 8759046 10.1037/0033-295x.103.3.518

[CR32] Harrell, F. E. (2024) Package ‘rms’ Version 6.8–1. Available on 06/18/2024 at https://cran.r-project.org/web/packages/rms/rms.pdf

[CR33] He, X., & Godfroid, A. (2019). Choosing words to teach: A novel method for vocabulary selection and its practical application. *TESOL Quarterly,**53*(2), 348–371.

[CR34] Heo, C. Y., Kim, B., Park, K., & Back, R. M. (2022). A comparison of Best-Worst Scaling and Likert Scale methods on peer-to-peer accommodation attributes. *Journal of Business Research,**148*, 368–377.

[CR35] Hollis, G. (2018). Scoring best-worst data in unbalanced many-item designs, with applications to crowdsourcing semantic judgments. *Behavior Research Methods,**50*(2), 711–729. 28550657 10.3758/s13428-017-0898-2

[CR36] Hollis, G. (2020). The role of number of items per trial in best–worst scaling experiments. *Behavior Research Methods,**52*(2), 694–722. 31290129 10.3758/s13428-019-01270-w

[CR37] Hollis, G., & Westbury, C. (2018). When is best-worst best? A comparison of best-worst scaling, numeric estimation, and rating scales for collection of semantic norms. *Behavior Research Methods,**50*(1), 115–133. 29322399 10.3758/s13428-017-1009-0

[CR38] Hulstijn, J. (2024). Predictions of individual differences in the acquisition of native and non-native languages: An update of BLC theory. *Languages,**9*(5), 173.

[CR39] James, W. (1890). The principles of psychology. Henry Holt.

[CR40] Jessen, F., Heun, R., Erb, M., Granath, D. O., Klose, U., Papassotiropoulos, A., & Grodd, W. (2000). The concreteness effect: Evidence for dual coding and context availability. *Brain and Language,**74*(1), 103–112. 10924219 10.1006/brln.2000.2340

[CR41] Johns, B. T. (2024). Determining the relativity of word meanings through the construction of individualized models of semantic memory. *Cognitive Science,**48*(2), Article e13413. 38402448 10.1111/cogs.13413

[CR42] Johnston, W. A., & Dark, V. J. (1986). Selective attention. *Annual Review of Psychology,**37*, 43–75. 10.1146/annurev.ps.37.020186.000355

[CR43] Juhasz, B. J. (2005). Age-of-acquisition effects in word and picture identification. *Psychological Bulletin,**131*(5), 684–712. 10.1037/0033-2909.131.5.684 16187854 10.1037/0033-2909.131.5.684

[CR44] Kassambara, A. & Patil, I. (2023). Package ‘ggcorrplot’, Version 0.1.4.1. Available on 06/18/2024 at https://cran.r-project.org/web/packages/ggcorrplot/ggcorrplot.pdf.

[CR45] Kornell, N. (2014). Attempting to answer a meaningful question enhances subsequent learning even when feedback is delayed. *Journal of Experimental Psychology: Learning, Memory, and Cognition,**40*(1), 106–114. 10.1037/a0033699 23855547 10.1037/a0033699

[CR46] Kuperman, V., Stadthagen-Gonzalez, H., & Brysbaert, M. (2012). Age-of-acquisition ratings for 30,000 English words. *Behavior Research Methods,**44*(4), 978–990. 22581493 10.3758/s13428-012-0210-4

[CR47] Lee, D. Y. (2001). Defining core vocabulary and tracking its distribution across spoken and written genres: Evidence of a gradience of variation from the British National Corpus. *Journal of English Linguistics,**29*(3), 250–278.

[CR48] Lee, C., & Gates, K. M. (2025). Automated machine learning for classification and regression: A tutorial for psychologists. *Behavior Research Methods,**57*, 262. 40826202 10.3758/s13428-025-02684-5

[CR49] Lewis, M. L., & Frank, M. C. (2016). The length of words reflects their conceptual complexity. *Cognition,**153*, 182–195. 27232162 10.1016/j.cognition.2016.04.003

[CR50] Logan, G. D. (1988). Toward an instance theory of automatization. *Psychological Review,**95*(4), 492–527. 10.1037/0033-295X.95.4.492

[CR51] Mandera, P., Keuleers, E., & Brysbaert, M. (2020). Recognition times for 62 thousand English words: Data from the English Crowdsourcing Project. *Behavior Research Methods,**52*, 741–760. 31368025 10.3758/s13428-019-01272-8

[CR52] Martínez, G., Molero, J. D., González, S., Conde, J., Brysbaert, M., & Reviriego, P. (2025). Using large language models to estimate features of multi-word expressions: Concreteness, valence, arousal. *Behavior Research Methods,**57*(1), 5. 10.3758/s13428-024-02515-z 39633225

[CR53] Mohammad, S. (2018, July). Obtaining reliable human ratings of valence, arousal, and dominance for 20,000 English words. In Proceedings of the 56th Annual Meeting of the Association for Computational Linguistics (pp. 174–184). https://aclanthology.org/P18-1017/

[CR54] Mohammad, S. M. (2026). From Trial by Fire To Sleep Like a Baby: A Lexicon of Anxiety Associations for 20k English Multiword Expressions. arXiv preprint arXiv:2602.18692.

[CR55] Monaghan, P., & Ellis, A. W. (2010). Modeling reading development: Cumulative, incremental learning in a computational model of word naming. *Journal of Memory and Language,**63*(4), 506–525.

[CR56] Muraki, E. J., Abdalla, S., Brysbaert, M., & Pexman, P. M. (2023). Concreteness ratings for 62,000 English multiword expressions. *Behavior Research Methods,**55*(5), 2522–2531. 35867207 10.3758/s13428-022-01912-6

[CR57] Pan, S. C., & Carpenter, S. K. (2023). Prequestioning and pretesting effects: A review of empirical research, theoretical perspectives, and implications for educational practice. *Educational Psychology Review,**35*(4), 97.

[CR58] Piantadosi, S. T., Tily, H., & Gibson, E. (2011). Word lengths are optimized for efficient communication. *Proceedings of the National Academy of Sciences,**108*(9), 3526–3529. 10.1073/pnas.1012551108 PMC3048148 21278332

[CR59] Rice, M. L., Buhr, J. C., & Nemeth, M. (1990). Fast mapping word-learning abilities of language-delayed preschoolers. *Journal of Speech and Hearing Disorders,**55*(1), 33–42. 2299838 10.1044/jshd.5501.33

[CR60] Rozin, P., Berman, L., & Royzman, E. (2010). Biases in use of positive and negative words across twenty natural languages. *Cognition and Emotion,**24*(3), 536–548.

[CR61] Rozin, P., & Royzman, E. B. (2001). Negativity bias, negativity dominance, and contagion. *Personality and Social Psychology Review,**5*(4), 296–320.

[CR62] Scott, G. G., Keitel, A., Becirspahic, M., Yao, B., & Sereno, S. C. (2019). The glasgow norms: Ratings of 5,500 words on nine scales. *Behavior Research Methods, 51*(3), 1258–1270. 10.3758/s13428-018-1099-3 PMC6538586 30206797

[CR63] Sendín, E., Conde, J., Reviriego, P., Haro, J., Ferré, P., Hinojosa, J. A., & Brysbaert, M. (2025). Combining the power of large language models with finetuning based on strategically collected human ratings: A case study about age-of-acquisition estimates of Spanish words. *Psicológica,**46*(2), Article e17563. 10.20350/DIGITALCSIC/17563

[CR64] Stubbs, M. (1986). Language development, lexical competence and nuclear vocabulary. Educational linguistics, 98–115.

[CR65] Van Ede, F., & Nobre, A. C. (2023). Turning attention inside out: How working memory serves behavior. *Annual Review of Psychology,**74*(1), 137–165. 35961038 10.1146/annurev-psych-021422-041757

[CR66] Van Heuven, W. J., Mandera, P., Keuleers, E., & Brysbaert, M. (2014). SUBTLEX-UK: A new and improved word frequency database for British English. *Quarterly Journal of Experimental Psychology,**67*(6), 1176–1190. 10.1080/17470218.2013.850521 24417251

[CR67] Wang, A., De Deyne, S., McKague, M., & Perfors, A. (2025). Core vocabulary in language representation and processing. *Cognitive Science*, *49*(12), e70151. 10.1111/cogs.70151 10.1111/cogs.70151 41370719

[CR68] Wang, A., De Deyne, S., McKague, M., & Perfors, A. (2026). Core vocabulary reveals differences between human word prediction and large language models. *Collabra: Psychology, 12*(1), 160142.

[CR69] Wei, T., Simko, V., Levy, M., Xie, Y., Jin, Y., & Zemla, J. (2017). Package ‘corrplot.’ *The Statistician,**56*(316), Article e24.

[CR70] Wickham, H. (2011). ggplot2. *Wiley interdisciplinary reviews: Computational statistics,**3*(2), 180–185.

[CR71] Wierzbicka, A. (1996). *Semantics: Primes and universals: Primes and universals*. Oxford University Press.

[CR72] Wundt, W. (1880). Grundzüge der physiologischen Psychologie [Principles of physiological psychology] (2nd ed., Vol. 1). Engelmann. http://vlp.mpiwg-berlin.mpg.de/references?id=lit575

[CR73] Yarkoni, T., Balota, D., & Yap, M. (2008). Moving beyond Coltheart’s N: A new measure of orthographic similarity. *Psychonomic Bulletin & Review,**15*(5), 971–979. 18926991 10.3758/PBR.15.5.971

[CR74] Zenner, E., Speelman, D., & Geeraerts, D. (2014). Core vocabulary, borrowability and entrenchment: A usage-based onomasiological approach. *Diachronica,**31*(1), 74–105.

[CR75] Zipf, G. K. (1936). *The psychobiology of language*. Routledge.

